# Competencies and clinical guidelines for managing acne with isotretinoin in general practice: a scoping review

**DOI:** 10.3399/BJGP.2025.0135

**Published:** 2025-08-12

**Authors:** Diarmuid Quinlan, Laura Sahm, Linda O’Keeffe, Miriam Santer, Alison M Layton, Tony Foley

**Affiliations:** 1 Department of General Practice, School of Medicine, University College Cork, Cork, Ireland; 2 medical director, Irish College of General Practitioners, Dublin, Ireland; 3 professor of clinical pharmacy, Pharmaceutical Care Research Group, School of Pharmacy, University College Cork, Cork, Ireland; 4 senior lecturer, School of Public Health, University College Cork, Cork, Ireland; 5 senior research fellow, MRC Integrative Epidemiology Unit, University of Bristol, Bristol, UK; 6 senior research fellow, Population Health Sciences, Bristol Medical School, University of Bristol, Bristol, UK; 7 professor of primary care research, Primary Care Research Centre, University of Southampton, Southampton, UK; 8 professor of dermatology, Skin Research Centre, Hull York Medical School, University of York, York, UK; 9 consultant dermatologist, Harrogate and District NHS Foundation Trust, Harrogate, UK; 10 professor of general practice, Department of General Practice, School of Medicine, University College Cork, Cork, Ireland

**Keywords:** acne vulgaris, clinical practice guideline, general practice, isotretinoin

## Abstract

**Background:**

Acne is a common, chronic, and burdensome disease. There is evidence of delayed and inequitable patient access to isotretinoin. Overuse of antibiotics in patients with acne raises antimicrobial stewardship concerns.

**Aim:**

To identify clinical practice guideline (CPG) and consensus statement recommendations regarding the clinical competencies required for prescribing oral isotretinoin for acne.

**Design and setting:**

This was a scoping review of acne CPGs and consensus statements, globally.

**Method:**

The Arksey and O’Malley framework informed design in conjunction with Joanna Briggs Institute guidance. The PRISMA extension for Scoping Reviews guided reporting. The search was conducted across six databases (Embase, Scopus, Web of Science, PubMed, CINAHL, PsycINFO), three guideline repositories (Scottish Intercollegiate Guidelines Network, Guidelines International Network, Trip), and grey literature. Two researchers independently screened titles and abstracts, and full-text papers. The AGREE II checklist appraised CPG quality.

**Results:**

From the initial 2292 articles, eight CPGs were included after applying inclusion and exclusion criteria. Five were from Europe, with one each from the US, Canada, and Malaysia. The CPG guidance varied regarding ‘Which doctor may prescribe isotretinoin?’ All CPGs indicated dermatologists and four identified GPs as appropriate prescribers. The CPGs identify the clinical competencies to safely manage people with acne using isotretinoin: dermatology, pregnancy prevention, mental health assessment, and blood testing.

**Conclusion:**

This scoping review has identified the key clinical competencies that underpin safe management of people with acne using isotretinoin: dermatology, pregnancy prevention, mental health assessment, and blood testing. Resourcing and supporting GPs to incrementally adopt this role may promote safe, timely, and equitable patient access to isotretinoin, while enhancing antimicrobial stewardship.

## How this fits in

There is evidence of inequitable access to the most effective treatment for severe acne, isotretinoin. This scoping review identified the clinical competencies to safely manage acne using isotretinoin. No global consensus exists among clinical practice guidelines (CGPs) on whether GPs are appropriate prescribers of isotretinoin. Appropriately resourced and CPG-guided patient access to isotretinoin in primary care may promote safe, timely, and equitable acne management for patients and improve antimicrobial stewardship.

## Introduction

Acne vulgaris (hereafter acne) is a chronic inflammatory disease of the pilosebaceous unit, mostly involving the face and torso, with comedones, seborrhoea, inflammatory papules, and pustules.^1^ Acne is common, affecting approximately 85% of teenagers and many adults, often with enduring scarring, hyperpigmentation, emotional, physical, educational, and psychosocial costs.^2–4^ Acne is the most common reason to visit a dermatologist.^4^ There is evidence of inequitable patient access to isotretinoin.^5–7^ Acne inflicts a fiscal burden on healthcare systems.^4,8^ Widespread, prolonged antibiotic use in acne raises antimicrobial stewardship concerns, emphasising the need for effective alternatives to antibiotics.^9–12^

Isotretinoin secured US Food and Drug Administration approval in 1982 for managing severe acne.^13^ Isotretinoin significantly improves acne by reducing the size, frequency, and severity of lesions, addressing the underlying aetiological pathways of the condition, and minimising the risk of long-term scarring.^13–20^ Although isotretinoin does not treat established scars, there is clear evidence that early effective treatment with isotretinoin results in less acne scarring.^21–23^ Isotretinoin has a well-documented profile of serious side effects, most specifically teratogenicity and blood dyscrasias, with concerns also raised about the impact on mental health and sexual function.^4,15,24,25^

Isotretinoin is highly teratogenic, with major congenital malformations affecting 15% of isotretinoin-exposed live births.^26^ Compliance with a robust Pregnancy Prevention Programme (PPP) among women taking isotretinoin has been reported as suboptimal in several countries.^27–30^ The isotretinoin PPP balances the competing priorities of protecting women’s access to isotretinoin while minimising foetal exposure.^31^ Timely provision of emergency contraception raises challenges for women and dermatologists.^32^

Isotretinoin has been associated with blood dyscrasias so regular blood testing and monitoring are commonly recommended.^15,16,18,33^ Clinically significant abnormal laboratory results are rare, especially among otherwise healthy people, raising the potential to reduce the number of some blood tests.^33^

The UK Commission on Human Medicines Isotretinoin Implementation Expert Advisory Working Group identified compelling case reports of sexual dysfunction and recommended age-appropriate patient counselling addressing *‘possible risk of sexual function side effects with isotretinoin*’.^34^ However, a recent scoping review concluded that the evidence of a causal relationship is ‘very poor’ and insufficient to substantiate such guidance.^35^

The mental health and emotional burdens of living with acne are well documented.^4,24^ Evidence suggests that treating acne with isotretinoin significantly improves symptoms of depression.^36^ There are concerns that isotretinoin is associated with neuropsychiatric side effects, including suicidality, mood changes, depression, and anxiety, requiring ongoing vigilance.^37^ The literature identifies individual case reports of adverse neuropsychiatric events, including mood change and depression among patients taking isotretinoin, and neurobiologists have provided an aetiological hypothesis.^37^ These significant concerns have perpetuated debate over many years and recently led to further regulatory review.^25^ The evidence from earlier regulatory reviews, and large database and cohort studies, has not identified a causal relationship linking adverse mental health and isotretinoin.^38^ A recent meta-analysis did not identify epidemiological evidence of an increased risk of suicide or depression among isotretinoin users at a population level.^39^

There is some evidence of ethnic group, sex, and social class disparities in acne care, with the underuse of isotretinoin among women, ethnic minority groups, and lower socioeconomic groups.^5–7^ Recognising healthcare disparities, isotretinoin regulations in New Zealand were amended in 2009.^6^ This extended state-subsidised isotretinoin to GP prescriptions, which historically had been restricted to dermatologists.^6^ GPs in New Zealand embraced prescribing isotretinoin, with 58% of isotretinoin prescriptions issued by GPs in 2012.^40^ The number of people in New Zealand taking isotretinoin increased yearly from 7709 people in 2006 to 23 983 people in 2023 (personal communication, Health New Zealand, 14 November 2024). In stark contrast, the introduction of the iPLEDGE, a risk evaluation and mitigation programme to minimise fetal exposure to isotretinoin, in the US in 2006 heralded a 30% reduction in isotretinoin prescriptions.^41^

There is considerable discussion about which doctor, GP, or dermatologist should initiate, prescribe, and monitor isotretinoin for patients with acne.^34,42^ Patient access to isotretinoin varies, with professional barriers restricting some clinicians from prescribing isotretinoin.^15,17^ Regulations in the UK enable prescribing by other clinicians working within a consultant dermatologist-supervised pathway, including junior hospital doctors, dermatology nurse specialists, and dermatology pharmacists.^34^ The UK regulations further recommend that, for adolescents <18years of age, a second healthcare professional should independently confirm isotretinoin as the most appropriate treatment.^34^

Clinical practice guidelines (CPGs) and consensus statements are widely used to standardise and enhance patient care.^43,44^ Recent review articles have examined important aspects of managing acne with isotretinoin.^45,46^ However, recommendations regarding competencies and which clinician should prescribe isotretinoin, have not been explored in detail. Globally, acne CPGs vary substantially, with some guidelines recommending restricting isotretinoin prescribing solely to dermatologists.^15,17^ Concern has been raised that this CPG prescribing restriction may adversely have an impact on timely and equitable patient access to isotretinoin.^31,46^ The severity of acne scarring is related to delays in effective acne treatment.^47,48^

Although all acne CPGs support the recommendation that dermatologists may initiate isotretinoin, the recommendations are more varied for GPs.^15–20,49^ The CPG recommendations regarding the key clinical competencies for managing patients with acne using isotretinoin have not been systematically examined.

This scoping review examined CPGs and consensus statements addressing acne management to explore and map the recommendations regarding the key clinical competencies required to safely manage people with acne using isotretinoin.

## Method

A scoping review of the literature was undertaken to address the study’s research questions. This scoping review was designed using the Arksey and O’Malley methodological framework along with the Joanna Briggs Institute (JBI) guidance.^50,51^ The reporting of the scoping review was guided by the PRISMA extension for Scoping Reviews (PRISMA-ScR).^51^ The scoping review protocol for this study was registered and published in PROSPERO and the full protocol was also published.^52,53^ The authors, assisted by a university medical librarian, developed the search strategy.

### Eligibility criteria

The most recent CPGs and consensus statements on acne management using oral isotretinoin, in any language, published between January 2013 and June 2024, were included. This timeframe captures current clinical practice. Scientific knowledge is in constant change and CPGs need to be up-to-date to maintain clinical validity.^54^ The authors diverged from the published protocol search strategy to include consensus statements (Box 1).^53^

**Box 1.** Eligibility criteria and search strategy

Eligibility criteria	
**Inclusion criteria**	**Exclusion criteria**
Most recent version of CPGs and consensus statements using isotretinoin for managing acne Published January 2013 to June 2024 AGREE II evaluation score >70% in four or more domains^57^ Any language	CPGs and consensus statements not addressing isotretinoin for managing acne Published before 2013 AGREE II evaluation score <70% in one, two, or three domains^57^ Case reports, editorials, conference proceedings, letters, local adaptations of clinical guidance, guidance addressing acne patient subgroups, research papers, opinion pieces
**Search strategy**	
**Keywords and synonyms:**	Acne Guideline OR guidance OR ‘best practice’ OR algorithm OR recommend*
**Databases**	Embase, Scopus, Web of Science, PubMed, CINAHL, and PsycINFO
**Google Scholar**	First 100 citations
**Citation search**	Hand-search citation list of all full papers extracted
**Guideline repositories**	SIGN, GIN, Trip
**Contact key informants**	Personal professional networks and all 20 EQuiP^55^ member countries

CPG = clinical practice guideline. EQuiP = European Society for Quality and Safety in Family Practice. GIN = Guidelines International Network. SIGN = Scottish Intercollegiate Guidelines Network.

### Search strategy

Two keywords and their synonyms: 1) acne 2) guideline OR guidance OR ‘best practice’ OR algorithm OR recommend* were used (Box 1).

Titles and abstracts were searched from six electronic databases (Embase, Scopus, Web of Science, PubMed, CINAHL, and PsycINFO), three guideline repositories (Scottish Intercollegiate Guidelines Network [SIGN], Guidelines International Network (GIN), and Trip), and Google Scholar in July 2024. The authors hand-searched the citation lists of all full papers.

Contacting key informants was undertaken. To identify restricted national guidance two authors (the first author and the senior author) engaged their professional networks in the US, UK, Canada, Australia, New Zealand, and the European Society for Quality and Safety in Family Practice (EQuiP),^55^ seeking additional relevant acne guidance. EQuiP emailed over 1700 EQuiP members.

#### Screening and study selection

Covidence reference management software was used to import and deduplicate references, screen titles, and abstracts, and undertake full-text review.^56^ Two reviewers (the first author and the senior author) independently screened all ‘titles and abstracts’ to identify eligible material for full paper review. Full papers that were unavailable in English were translated using Google document translate. The text of full papers was independently evaluated by two reviewers (the first author and the senior author) against the eligibility criteria. A third team member was available but not required to achieve consensus.

**Figure 1.**
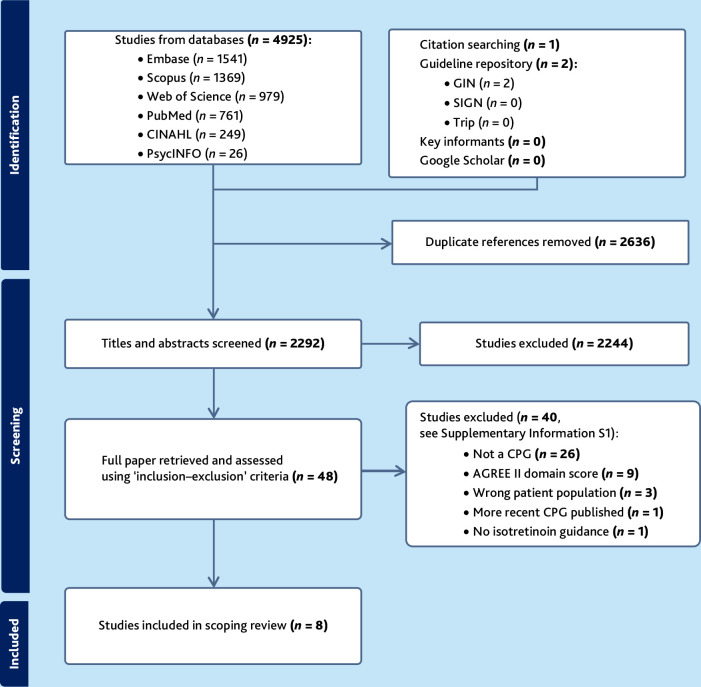
PRISMA flowchart. CPG = clinical practice guideline. GIN = Guidelines International Network. SIGN = Scottish Intercollegiate Guidelines Network.

#### Quality appraisal of CPGs and consensus statements

Guideline quality was objectively appraised using the AGREE II checklist and calculated for the six domain scores of potentially relevant guidelines.^57^ One author (the first author) evaluated and scored the guidelines against the full AGREE II checklist, with verification by another reviewer (the senior author).^57^ This was an iterative process. The guidelines included in this scoping review have AGREE II scores >70% across four, five, or six domains, in line with inclusion criteria in previous studies.^46,58^

#### Data extraction

A data extraction tool was developed using Microsoft Excel, modelled on the JBI data extraction tool, and adapted on an iterative basis.^59^ The extracted data items are presented in the results section. One reviewer (the first author) extracted and another (the senior author) verified the data items from the eight CPGs. Where clarification was necessary, one reviewer (the first author) contacted the guideline author.

## Results

The searches identified 4928 articles. Following deduplication, 2292 articles were screened using title and abstract by two reviewers (the first author and the senior author) A full-text review of 48 articles was undertaken by two reviewers (the first author and the senior author), with eight articles included in the scoping review. The 40 excluded full-text papers and the reasons for exclusion are displayed in Supplementary Information S1. Sixty key informants responded without identifying any additional acne clinical guidance (Figure 1).

### Key characteristics of included CPGs

#### CPG country of origin, author team, and target clinical audience

Five CPGs originated from Europe,^18–20,49,60^ with one each from the US,^15^ Canada,^16^ and Malaysia (Table 1).^17^ All CPG author teams included dermatologists. GPs contributed to the authorship of five CPGs.^17,18,20,49,60^ The author team of the UK and French CPGs are especially multidisciplinary.^20,49^ Two CPGs included a patient voice.^15,20^ One CPG was informed by a patient focus group and a review of acne literature addressing the patient’s perspective.^18^

**Table 1.** Overview of included CPGs

Country	CPG author team	Target clinical audience	‘Which doctor may prescribe isotretinoin?’
Netherlands^18^	***N*** **= 8** Dermatologist: 1 GP: 7 Patient focus group	GPs	Dermatologist GP
US^15^	***N*** **= 14** Dermatologist: 12 One each: patient representative, staff liaison	Dermatologists	Dermatologist
UK, NICE^20^	***N*** **= 15** Dermatologist: 4 GP: 2 Lay member: 2 One each: pharmacist, dermatology specialist nurse, psychiatrist, psychologist, dietician, microbiologist, gynae-endocrinologist	‘HCPs providing NHS-commissioned services’	Dermatologist and GP under dermatologist governance
Malaysia^17^	***N*** **=14** Dermatologist: 8 GP: 2 One each: public health doctor, dietician, pharmacist, HTA staff	‘Medical professionals, allied health professionals, trainees and medical students, professional societies and policy makers AND in primary, secondary and tertiary healthcare settings’	Dermatologist
France^49^	***N*** **= 19** Dermatologist: 7 GP: 2 Methodologist: 2 Gynaecologist: 2 One each: drug-safety specialist, endocrinologist, ID specialist, psychiatrist, paediatrician, microbiologist	Not addressed	Not addressed
Belgium^60^	***N*** **= 4** Dermatologist: 1 GP: 2 Pharmacist: 1	GPs	Dermatologist GP
Europe^19^	***N*** **= 18** Dermatologist: 18	Not addressed	Not addressed
Canada^16^	***N*** **= 11** Dermatologist: 11	GPs, dermatologists, nurses, pharmacists, paediatricians, obstetricians/gynaecologists	Dermatologist GP

CPG = clinical practice guideline. HCP = healthcare professional. NICE = National Institute for Health and Care Excellence. ID = infectious diseases specialist. HTA = Malaysian Health Technology Assessment Section, Ministry of Health.

The target clinical audience of CPGs varies. The Netherlands^18^ and Belgian^60^ CPGs were written specifically for GPs. Dermatologists were the target audience of the US CPG.^15^ The target clinical audiences for the UK,^20^ Canada,^16^ and Malaysia CPGs^17^ were broad.

#### CPG recommendations: ‘Which doctor may prescribe isotretinoin?’

The CPG recommendations vary considerably regarding which doctor may prescribe isotretinoin (Table 1). Three CPGs support GPs to independently prescribe isotretinoin (Canada, Netherlands, Belgium).^16,18,60^ In contrast, the UK National Institute for Health and Care Excellence (NICE) guidance supports GPs but under the governance of a consultant dermatology-led service.^20^

#### CPG recommendations: isotretinoin and pregnancy prevention

Seven CPGs offer recommendations regarding a mandatory PPP^15–18,20,49,60^ and one CPG omits this issue^19^ (Table 2).

**Table 2.** Recommendations regarding isotretinoin and pregnancy prevention

Country	Isotretinoin and PPP			
	Mandatory PPP?	What PPP?	Prescribed contraception mandatory if not sexually active?	Pregnancy test undertaken by whom?
Netherlands^18^	Yes	Dual contraception	Contraception not required	GP
US^15^	Yes	Dual contraception	Contraception not required ‘but must adhere to complete abstinence’	Not specified
UK, NICE^20^	Yes	Dual contraception	Contraception not required ‘No sexual activity’^34^	Not specified
Malaysia^17^	Yes	Not specified but ‘strict contraceptive’	Ambivalent: ‘strict contraceptive practice is required for females who may become pregnant’	Not specified
France^49^	Yes	Not specified but ‘notably, prevention of pregnancy ... mandatory’	Contraception not required	Not specified
Belgium^60^	Yes	Not addressed	Contraception not required	GP
Europe^19^	Not addressed	Not addressed	Not addressed	Not specified
Canada^16^	Yes	Not specified but ‘pregnancy prevention programs … are essential’	Not addressed	Not specified

NICE = National Institute for Health and Care Excellence. PPP = pregnancy prevention programme.

Dual contraception is recommended in three CPGs.^15,18,20^ Three CPGs recommend a robust PPP but do not specify dual contraception.^16,17,19,49,60^

Five CPGs acknowledge that, while a robust PPP is required for all women of childbearing potential, contraception is not mandatory for those women who are not sexually active.^15,18,20,49,60^ The Malaysia CPG is less specific.^17^

#### CPG recommendations: isotretinoin and mental health assessment

Seven CPGs recommend regular and periodic mental health assessments for people taking isotretinoin (Table 3).^15–18,20,49,60^ Three CPGs support the use of screening tools to assess mental health.^15,20,49^ One CPG acknowledged ‘safety concerns’ without recommending mental health assessment.^19^ Two CPGs recommend that GPs undertake the mental health assessment^18,60^ and one CPG recommends ‘the treating clinician’^16^. The recommended frequency of mental health assessments varies considerably across CPGs (Table 3).

**Table 3.** Recommendations regarding isotretinoin and mental health assessment

Country	Routine mental health assessment			
	Undertaken by which clinician?	Assessment for which conditions?	Screening tool recommended? Which tool?	Frequency of assessment?
Netherlands^18^	GP	‘Depression or psychosis’	Not addressed	‘Every 4 weeks’
US^15^	Not specified	‘Depression, anxiety, suicidal ideation/suicidality and other neuropsychiatric side effects’	Yes: PHQ-2, PHQ-9	Not specified
UK, NICE^20^	Not specified	‘Psychological wellbeing’	Yes,^a^ consider: PHQ-9, GAD-7, GAD-2, HADS, PHQ-A	‘Monitor regularly’
Malaysia^17^	Not specified	Depression	Not addressed	‘Before and during treatment’
France^49^	Not specified	Depression, mood, or behaviour change	Yes: Adolescent Depression Rating Scale	Not specified
Belgium^60^	GP	Depression	Not addressed	‘Assessed before and during treatment’
Europe^19^	Not specified	Not addressed	Not addressed	Not specified
Canada^16^	*‘*The treating physician’	*‘*Monitor for signs and symptoms of psychiatric disturbance*‘*	Not addressed	*‘*Evaluate monthly’

a ‘There are no validated screening tools … for assessment of mental health in the context of prescribing isotretinoin for acne.’^34^ *GAD = General Anxiety Disorder. HADS = Hospital Anxiety and Depression Scale. NICE = National Institute for Health and Care Excellence. PHQ = Patient Health Questionnaire. PHQ-A = PHQ Adolescent.*

#### CPG recommendations: isotretinoin and blood testing

Blood testing is recommended in five CPGs (Table 4).^15,17,18,20,60^ There is heterogeneity in the recommended blood tests and testing frequency.

**Table 4.** Recommendations regarding isotretinoin and blood testing

Country	Blood testing			
	Recommend and frequency?	Liver function	Lipids	Full blood count
Netherlands^18^	Yes, 0, 1, 4 months	Yes	Yes	Yes
US^15^	Yes, frequency not indicated	Yes	Yes	Not indicated
UK, NICE^20^	Yes, as per MHRA, 0, 1, 3 months	Yes, as per MHRA	Yes, as per MHRA	No, as per MHRA
Malaysia^17^	Yes, 0 and 6–8 weeks	Yes	Yes	Not indicated
France^49^	Not addressed	Not addressed	Not addressed	Not addressed
Belgium^60^	Yes, 0, 1, and every 3 months	Yes	Yes	Yes
Europe^19^	Not addressed	Not addressed	Not addressed	Not addressed
Canada^16^	Not addressed	Not addressed	Not addressed	Not addressed

MHRA = Medicines and Healthcare products Regulatory Agency. NICE = National Institute for Health and Care Excellence.

## Discussion

### Summary

This is the first scoping review, to the authors’ knowledge, to explore in detail CPG recommendations for the key clinical competencies required to safely manage acne using isotretinoin. This review highlights strategic clinical issues, including patient safety, equity of access, and antimicrobial stewardship. This scoping review identifies many consistent and some diverging CPG recommendations to enhance the safe use of isotretinoin, especially a mandatory PPP, mental health assessment, and blood monitoring. Differing recommendations were found regarding which clinician may prescribe isotretinoin, for instance within the neighbouring countries of US and Canada, whereas in other jurisdictions a consensus was found, for instance, between neighbouring Belgium and the Netherlands.

### Strengths and limitations

A strength of this scoping review is the international multidisciplinary research team comprising a dermatologist, GPs, and academics. An extensive search process was undertaken without language restrictions and two GP authors independently reviewed all academic material. Guideline quality was objectively appraised using the AGREE II checklist and included guidelines that met a defined CPG quality standard. The study excluded, and hence does not report, findings from guidelines that did not meet these AGREE II criteria. However, those excluded guidelines may be widely used in clinical practice internationally, hence some areas of clinical practice around isotretinoin and acne management may be underreported.

### Comparison with existing literature

There is considerable evidence of inequitable access to isotretinoin for women, ethnic minority communities, and socially disadvantaged people.^5–7,61^ Inequitable access to isotretinoin in New Zealand catalysed policy change.^6,61^ A single policy intervention at the point of prescribing incrementally expanded access to isotretinoin in New Zealand.^61^ Furthermore, for patients with severe acne unresponsive to combination therapy, timely access to isotretinoin in primary care may address two strategic concerns: minimising acne scarring and enhancing antimicrobial stewardship.

This scoping review identified strategic CPG consistencies, most notably the mandatory PPP recommended in seven of eight CPGs.^15–18,20,49,60^ However, there are diverging CPG recommendations regarding single–dual contraception and contraception for women who are not sexually active. Dermatologists in the US have identified challenges in managing complex sexual health issues.^32^ The most common reasons for delayed initiation and premature termination of isotretinoin therapy in the US relate to onerous iPledge PPP regulations.^5,7^

National policy may have an impact on patient access to isotretinoin. The New Zealand experience of amending national policy to dismantle such barriers merits wider consideration. The UK Commission on Human Medicines (CHM) suggested that *‘the potential for GPs with extended roles to independently prescribe isotretinoin for adult patients should be explored … and reflected in clinical guidance’*.^34^

This current review identified that most CPGs recommend regular mental health assessments for people taking isotretinoin. However, the CPGs diverge on clinically relevant issues, including which clinician should undertake this assessment, the clinical role of mental health screening tools, the frequency, and relevant mental health conditions. Clinicians prescribing isotretinoin should be skilled in mental health assessment.^39^

This current review identified considerable heterogeneity around blood testing recommendations. The CPGs seem to be at odds with the likelihood ratios, and blood testing may unnecessarily consume limited healthcare resources.^33^ Notwithstanding these concerns, blood monitoring of isotretinoin, where appropriate, is more accessible for patients and more cost-effective for healthcare systems in primary care.

### Implications for research and practice

The safe, equitable, and timely management of acne using isotretinoin has policy, practice, and research implications for all stakeholders.^62,63^ This scoping review highlights differing CPG recommendations around the GP role for PPP, mental health assessment, and blood monitoring. Safe use of isotretinoin is of paramount importance, requiring broad clinical expertise across dermatology, sexual health, mental health, and medicines management. Inequitable access to isotretinoin disadvantages many patients and should be a key priority for policymakers. Timely access to isotretinoin promises enhanced patient outcomes and may enhance antimicrobial stewardship.

There is a considerable global deficit of healthcare professionals, including GPs and dermatologists.^62,63^ This workforce deficit adversely has an impact on timely patient access to holistic GP care. Long waiting times for specialist hospital care contribute to patient dissatisfaction and increase demands on primary care. Managing acne with isotretinoin is resource intensive. Migration of this clinical role to GPs is a paradigm shift. The New Zealand experience, where GPs with appropriate acne education and support have absorbed the incremental isotretinoin workload, makes a compelling case to explore broader implementation.^61^ Further research is needed on timely patient access, safety, feasibility, acceptability, resourcing, and workload implications of GPs prescribing isotretinoin. A national policy decision to adequately support and resource GPs is fundamental to underpin safe and sustainable migration of this expanded dermatology role to general practice. Although data from New Zealand are encouraging, the wider health system implications of such reform will require considerable research to capture the perspectives of GPs, dermatologists, and patients.

In conclusion, this scoping review has identified the diverse clinical competencies that underpin the safe management of people with acne using isotretinoin: dermatology, pregnancy prevention, medicines management, and mental health. Appropriately resourced and CPG -guided patient access to isotretinoin in primary care may enhance safe, timely, and equitable acne management for patients and improve antimicrobial stewardship.

## Data

The research papers on which this research relies are all publicly available with the datasets shown in the results section and as supplementary data.
